# Associations between SII, SIRI, and cardiovascular disease in obese individuals: a nationwide cross-sectional analysis

**DOI:** 10.3389/fcvm.2024.1361088

**Published:** 2024-08-22

**Authors:** Zhou Liu, Longxuan Zheng

**Affiliations:** Department of Cardiology, Huai’an Hospital Affiliated to Yangzhou University (The Fifth People's Hospital of Huai’an), Huai’an, China

**Keywords:** systemic immune-inflammation index, systemic inflammation response index, cardiovascular diseases, inflammation, obesity, prevalence

## Abstract

**Background:**

Systemic immune-inflammation index (SII) and systemic inflammation response index (SIRI) are comprehensive markers of inflammatory status. However, the correlation between SII and SIRI and the prevalence of cardiovascular disease (CVD) in populations with obesity remains unknown.

**Methods:**

This is a cross-sectional study with data obtained from the National Health and Nutrition Examination Survey from 1999 to 2018. SII and SIRI were calculated using the following equations: SII = (platelet count × neutrophil count)/lymphocyte count. SIRI = (neutrophil count × monocyte count)/lymphocyte count. Spearman’s rank correlation coefficient was used to assess the relationship between SII and SIRI and baseline variables. Logistic regression models and generalized additive model (GAM) with a spline smoothing function were used to evaluate the association between SIRI and CVD prevalence. Nomogram and receiver operating characteristic curve (ROC) analysis were used to assess the value of the risk prediction model.

**Results:**

A total of 17,261 participants with obesity and SII and SIRI publicly available data were used for this study. Multivariate logistic regression analysis revealed that SIRI, rather than SII, was an independent risk factor for CVD prevalence. For every standard deviation increase in SIRI, there was a 13%, 15%, and 28% increase in the odds ratios of CVD prevalence (OR = 1.13, 95% CI: 1.04–1.22, *P* = 0.01), coronary heart disease (OR = 1.15, 95% CI: 1.05–1.26, *P* = 0.002), and congestive heart failure (OR = 1.28, 95% CI: 1.16–1.41, *P* < 0.001). ROC results demonstrated that SIRI had a certain accuracy in predicting CVD prevalence (AUC = 0.604), especially when combined with other variables used in the nomogram (AUC = 0.828). The smooth curve fitting regression analysis demonstrated a significant linear association between the risk of SIRI and the odds ratio of CVD prevalence (*P* for nonlinear = 0.275).

**Conclusions:**

SIRI is a relatively stable indicator of inflammation and is independently associated with the prevalence of CVD. It may serve as a novel inflammatory indicator to estimate CVD prevalence in populations with obesity.

## Introduction

The increasing prevalence of obesity (BMI ≥ 30 kg/m^2^) is a serious public health concern worldwide. In the United States, the prevalence of obesity in adults has risen from 30.5% to 42.4% over the past two decades, with nearly 10% classified as severely obese (BMI ≥ 40 kg/m^2^) (1). Elevated adipokines levels, secreted by adipocytes trigger a chronic inflammatory response that can lead to various chronic systemic diseases, including heart attack, stroke, nonalcoholic fatty liver disease (NAFLD), diabetes mellitus (DM), and multiple cancer types (2–4). Consequently, obesity and its related conditions have significant physical and economic impacts, placing an additional burden on individuals and health insurance systems (5, 6).

Although previous studies have reported that several indicators (e.g., vitamin D, interleukin- 6 [IL-6], C-reactive protein [CRP]) could reflect inflammation levels and affect the prognosis of chronic diseases (7–10), using single or a few inflammatory makers are insufficient to assess the overall inflammatory state of the body since these markers may have anti- or pro-inflammatory functions in different biological processes (11). Moreover, composite inflammatory indicators evaluated using different hematological parameters are more representative of the overall inflammatory state.

Systematic immune inflammation index (SII) and systemic inflammation response index (SIRI) are novel markers of inflammatory diseases (12). These two indexes used neutrophil counts and lymphocyte counts respectively. The calculation formula for SII is (plateletcount×neutrophilcount)/lymphocytecount, and for SIRI,itis(neutrophilcount×monocytecount)/lymphocyte count. The principle behind these new indexes is based on the roles of different types of blood cells in the inflammatory response, comprehensively reflecting the state of immunity and inflammation (13). Recent studies have explored the value of SII and SIRI in predicting cardiovascular events (13, 14). Obese individuals have a higher risk of cardiovascular disease (CVD) due to metabolic disorders and more intense inflammatory responses (2). However, the association between SII and SIRI and the prevalence of CVD in individuals with obesity remains unclear. In this study, we comprehensively investigated the relationship between SII and SIRI with the prevalence of CVD in obese patients.

## Methods

### Study design and population

This cross-sectional study utilized data from the National Health and Nutrition Examination Survey (NHANES) conducted between 1999 and 2018. NHANES is a survey of the civilian non-institutionalized population in the United States that uses a complex stratified multistage probability design (15). The survey includes interviews, physical examinations [at home or a mobile examination center (MEC)], and laboratory tests and is conducted every 2 years. During the period 1999–2018, NHANES conducted 10 separate cycles. Each cycle included entirely new participants, and each participant had a unique identification number, ensuring the uniqueness of the data. Therefore, we could determine that there was no overlap of participants between cycles. In addition, when participants were included in a particular cycle, their baseline clinical characteristics were interviewed or collected during that cycle. NHANES was conducted by the National Center for Health Statistics of the Centers for Disease Control and Prevention (CDC) and approved by the NHANES Institutional Review Board, with written informed consent obtained from all participants.

A total of 23,480 adults (age >18 years), who were classified as obese, were included in the NHANES survey between 1999 and 2018. Of these, 3,997 participants were excluded due to the inability to calculate SII or SIRI data. Of the remaining 19,483 participants, 542 pregnant women and 1,680 cancer patients were excluded, leaving 17,261 obese adults for the analysis.

### Definition of SII and SIRI

The Beckman Coulter MAXM instrument at the MEC produced complete blood cell counts on blood specimens. Detailed specimen collection and processing instructions are available in the Laboratory/Medical Technologists Procedures Manual (16).

SII and SIRI were calculated using the following formulas (17, 18):

SII = (platelet count × neutrophil count)/lymphocyte count
SIRI = (neutrophil count × monocyte count)/lymphocyte count

### Definition and diagnosis of CVD

CVD is a composite of a group of specific cardiovascular diseases, including heart attack, angina, congestive heart failure, coronary heart disease, and stroke (19). The diagnosis of specific CVD was based on self-reported medical history in the medical conditions section of NHANES. The primary outcome of this study is the prevalence of CVD. The secondary outcome is the prevalence of five specific CVDs, including heart attack, angina, congestive heart failure, coronary heart disease, and stroke.

### Other variables of interest

Standardized questionnaires were used to collect information on age, gender, ethnicity, education level, family income, smoking and drinking behavior, medical history, and medication use. Medical history was based on self-reported previous medical records from a healthcare professional or a physician. Biochemical parameters were measured using a rigorous procedure, the details of which are provided in the NHANES Procedures Manual for Laboratory/Medical Technologists (16). To facilitate data integration, the following variables were further classified:

(i) Ethnicity: Non-Hispanic white, non-Hispanic black, Mexican American, or other ethnicities
(ii) Educational level: Less than 9th grade, 9–11th grade/high school or equivalent, college graduate, or above
(iii) Smoking status: Never (<100 cigarettes/lifetime), former smoker (≥100 cigarettes/lifetime and currently not smoking), or current smoker (>100 cigarettes/lifetime and currently smoking some days or every day) (20)
(iv) Drinking status: Never (<12 drinks/lifetime), former drinker (≥12 drinks/lifetime but not in the past year), current light/moderate drinker (≤1 drink/day for women and ≤2 drinks/day for men in the past year), or current heavy drinker (>1 drink/day for women and >2 drinks/day for men in the past year) (21)

### Statistical analysis

To provide nationally representative estimates, MEC weights were utilized in data analysis to account for oversampling, non-response, and non-coverage. Details on weighting methods are available on the NHANES website (https://wwwn.cdc.gov/nchs/nhanes).

Baseline demographic characteristics were presented as means (standard error, SE) and continuous variables and weighted percentages (95% confidence interval, CI) for categorical variables. Spearman's rank correlation coefficient was used to determine the association between SII and SIRI and baseline variables, including age, BMI, alanine aminotransferase (ALT), aspartate aminotransferase (AST), total cholesterol (TC), high-density lipoprotein cholesterol (HDL), estimated glomerular filtration rate (eGFR), and medical history. Quartiles were used to categorize the level of SII and SIRI as categorical variables. A standard normal distribution of SII and SIRI [mean = 0, standard deviation (SD) = 1] was created by standardizing the *Z*-scores.

Multivariate logistic regression models were used to calculate odds ratios (ORs) and 95% CIs between SII and SIRI levels (quartiles and per-SD) and CVD prevalence. Baseline variables were considered as candidate predictors for the multivariate regression model. Confounding covariates were progressively added to different models. Subgroup analyses were performed stratified by clinical characteristics, including sex (male or female), age (<65 or ≥65 years), body mass index (30–34.9, 35–39.9, or ≥40 kg/m^2^), ethnicity (non-Hispanic white, non-Hispanic black, Mexican American, and others), DM (no or yes), hypertension (no or yes), hyperlipidemia (no or yes), and asthma (no or yes). In subgroup analyses, the effect of SIRI level on the odds ratio of CVD prevalence with each 1 SD increase was assessed. Meanwhile, interaction *P* values between each stratification variable (e.g., male/female) were calculated to examine the consistency of the trend toward CVD prevalence with each 1 SD increase in SIRI level. If an interaction *p*-value of less than 0.05 is observed, this suggests that the association between SIRI and CVD prevalence in this stratification is significantly different across subgroups, with the association likely to be more pronounced in one subgroup than the others.

A generalized additive model (GAM) with a spline smoothing function was used to assess the dose-response relationship between SIRI levels and CVD prevalence. Nonlinear *P*-values were obtained using the log-likelihood ratio test (22). If nonlinear *P*-value is less than 0.05, the fitted curve is nonlinear and vice versa. For nonlinear association, threshold effect analysis based on a two-piecewise linear regression model was performed to calculate the inflection point in the smoothing curve at which the relationship between SIRI and CVD prevalence changed significantly (23).

Nomograms were constructed for clinical applications using logistic regression models. Variables were screened using univariate analysis and multivariate logistic regression. The discriminative power of the model was assessed using the area under the curve (AUC) and calibration of risk predictions via the bias-corrected calibration intercept (i.e., predicted vs. observed outcome) (24). Furthermore, decision curve analysis (DCA) was performed by estimating the net benefit at different threshold probabilities to determine the suitability of the established column line graphs for clinical application (25).

All analyses were conducted using the statistical packages R (version 4.2.1, R Foundation) and EmpowerStats (version 4.2.0, www.R-project.org, X&Y Solutions, Inc., Boston, MA). Bilateral *P*-values less than 0.05 were considered statistically significant.

## Results

### Clinical baseline characteristics of participants with obesity

A total of 17,261 participants with obesity were included, representing 68,648,287 individuals. The average age of the participants was 48.3 years, and 44.9% were male. Participants with obesity were divided into Q1, Q2, Q3, and Q4 based on SII and SIRI values. Significant differences were found in age, poverty income ratio (PIR), BMI, HDL, TC, ALT, AST, gender, ethnicity, education levels, DM, hyperlipidemia, smoking, and antihypertensive use (all *P* < 0.05) for SII. Similarly, for SIRI, significant differences were observed between participants with higher and lower SIRI values in age, PIR, BMI, HDL, TC, ALT, gender, ethnicity, education levels, DM, hypertension, smoking, drinking, antihypertensive use and glucose-lowering drug use (all *P* < 0.05). The detailed results of baseline characteristics of participants with obesity are presented in Supplementary Table S1 (for SII) and Table 1 (for SIRI).

**Table 1:** Survey-weighted baseline characteristics of the obese population in NHANES from 1999 to 2018 according to SIRI quartiles (*N* = 17,261, representing 68,648,287 individuals with obesity).

	Q1 (<0.72)	Q2 (0.72–1.07)	Q3 (1.07–1.53)	Q4 (>1.53)	*P*-value
Participants number	4,304	4,314	4,327	4,316	
Representing sample size	14,317,038	17,482,015	18,338,877	18,510,357	
Age (years)	45.12 (0.30)	45.59 (0.29)	46.90 (0.33)	48.52 (0.34)	<0.001
PIR	2.72 (0.04)	2.93 (0.04)	2.94 (0.04)	2.79 (0.04)	<0.001
BMI(Kg/m^2^)	35.41 (0.12)	35.52 (0.11)	35.84 (0.11)	36.66 (0.15)	<0.001
HDL (mmol/L)	1.27 (0.01)	1.23 (0.01)	1.21 (0.01)	1.21 (0.01)	<0.001
TC (mmol/L)	5.18 (0.02)	5.11 (0.02)	5.10 (0.02)	4.98 (0.03)	<0.001
eGFR	98.48 (0.49)	96.22 (0.49)	94.33 (0.46)	91.80 (0.52)	<0.001
ALT (U/L)	27.45 (0.38)	28.94 (0.35	29.20 (0.47)	29.11 (0.44)	0.011
AST (U/L)	25.17 (0.23)	25.50 (0.24)	25.58 (0.33)	25.86 (0.34)	0.374
Gender					<0.001
Female	61.37 (59.25, 63.44)	55.38 (53.35, 57.39)	50.43 (48.50, 52.36)	46.20 (43.93, 48.49)	
Male	38.63 (36.56, 40.75)	44.62 (42.61, 46.65)	49.57 (47.64, 51.50)	53.80 (51.51, 56.07)	
Ethnicity					<0.001
Non-Hispanic white people	47.18 (43.62, 50.77)	64.26 (61.46, 66.96)	69.08 (66.15, 71.86)	73.58 (70.86, 76.14)	
Non-Hispanic black people	31.03 (27.99, 34.23)	13.60 (12.00, 15.37)	9.86 (8.55, 11.33)	7.77 (6.69, 9.00)	
Mexican American	10.85 (9.27, 12.66)	11.04 (9.52, 12.76)	10.22 (8.65, 12.03)	8.69 (7.23, 10.42)	
Other Ethnicities	10.95 (9.55, 12.51)	11.11 (9.78, 12.59)	10.85 (9.48, 12.38)	9.96 (8.55, 11.56)	
Education levels					0.001
Less than 9th grade	7.10 (6.30, 7.99)	6.42 (5.69, 7.24)	5.45 (4.70, 6.31)	5.88 (5.06, 6.82)	
9–11th grade/high school grade or equivalent	37.94 (35.98, 39.95)	36.04 (34.01, 38.12)	39.47 (37.40, 41.57)	41.16 (39.00, 43.35)	
College graduate or above	54.96 (52.94, 56.97)	57.54 (55.36, 59.69)	55.08 (52.83, 57.30)	52.96 (50.72, 55.19)	
DM					<0.001
No	82.54 (81.06, 83.93)	82.24 (80.86, 83.54)	79.81 (78.23, 81.29)	74.47 (72.68, 76.18)	
Yes	17.46 (16.07, 18.94)	17.76 (16.46, 19.14)	20.19 (18.71, 21.77)	25.53 (23.82, 27.32)	
Hyperlipidemia					0.076
No	21.74 (20.07, 23.51)	21.40 (19.70, 23.20)	19.36 (17.90, 20.91)	19.42 (17.74, 21.22)	
Yes	78.26 (76.49, 79.93)	78.60 (76.80, 80.30)	80.64 (79.09, 82.10)	80.58 (78.78, 82.26)	
Hypertension					<0.001
No	55.14 (52.97, 57.28)	55.65 (53.46, 57.81)	51.20 (49.16, 53.23)	44.83 (42.95, 46.73)	
Yes	44.86 (42.72, 47.03)	44.35 (42.19, 46.54)	48.80 (46.77, 50.84)	55.17 (53.27, 57.05)	
Asthma					0.303
No	88.23 (87.01, 89.35)	86.38 (84.85, 87.79)	87.22 (85.78, 88.54)	87.22 (85.83, 88.49)	
Yes	11.77 (10.65, 12.99)	13.62 (12.21, 15.15)	12.78 (11.46, 14.22)	12.78 (11.51, 14.17)	
Smoking					<0.001
Never	62.13 (60.34, 63.88)	59.14 (57.05, 61.19)	52.90 (50.92, 54.87)	48.47 (46.28, 50.67)	
Former	21.23 (19.54, 23.02)	24.20 (22.33, 26.17)	27.10 (25.33, 28.95)	28.61 (26.75, 30.54)	
Current	16.64 (15.13, 18.28)	16.66 (15.38, 18.03)	20.00 (18.34, 21.78)	22.92 (21.32, 24.60)	
Drinking					0.038
Never	13.57 (11.82, 15.53)	13.10 (11.50, 14.88)	12.41 (10.89, 14.10)	10.83 (9.60, 12.21)	
Former	17.02 (15.25, 18.96)	15.78 (14.16, 17.54)	16.45 (14.91, 18.11)	19.26 (17.55, 21.09)	
Mild/moderate	32.74 (30.50, 35.06)	32.86 (30.62, 35.17)	34.10 (31.94, 36.33)	33.02 (30.97, 35.14)	
Heavy	36.67 (34.64, 38.76)	38.27 (36.30, 40.27)	37.05 (34.88, 39.27)	36.89 (34.77, 39.07)	
Antihypertensives					0.003
No	89.80 (88.45, 91.00)	89.78 (88.25, 91.14)	88.51 (87.23, 89.67)	86.42 (85.05, 87.67)	
Yes	10.20 (9.00, 11.55)	10.22 (8.86, 11.75)	11.49 (10.33, 12.77)	13.58 (12.33, 14.95)	
Glucose-lowering drugs					<0.001
No	90.12 (88.93, 91.20)	89.05 (87.96, 90.05)	87.27 (85.84, 88.57)	83.07 (81.60, 84.44)	
Yes	9.88 (8.80, 11.07)	10.95 (9.95, 12.04)	12.73 (11.43, 14.16)	16.93 (15.56, 18.40)	

Categorical variables were expressed as survey-weighted percentage (95% confidence interval).

Continuous variables were expressed as survey-weighted mean (standard Error, SE).

PIR, poverty income ratio; BMI, body mass index; ALT, alanine aminotransferase; DM, diabetes mellitus; AST, aspartate aminotransferase; TC, total cholesterol; HDL, high-density lipoprotein cholesterol; CVD, cardiovascular diseases.

### Correlation between SII, SIRI, and CVD prevalence

Spearman correlation analysis revealed that, for SII, the prevalence of CVD was negatively correlated with age [Spearman correlation coefficient (rho) = −0.05, *P* < 0.001], gender (rho = −0.10, *P* < 0.001), ethnicity (rho = −0.06, *P* < 0.001), ALT (rho = −0.09, *P* < 0.001), and AST (rho = −0.13, *P* < 0.001). However, the prevalence of CVD was positively correlated with BMI (rho = 0.1, *P* < 0.001) and hyperlipidemia (rho = 0.03, *P* < 0.001). Moreover, the prevalence of CVD was not statistically correlated with the variables, including HDL (*P* = 0.31), TC (*P* = 0.21), eGFR (*P* = 0.09), DM (*P* = 0.14), hypertension (*P *= 0.54), and asthma (*P* = 0.70). Additionally, for SIRI, CVD prevalence was negatively correlated with ethnicity (rho = −0.12, *P* < 0.001), HDL (rho = −0.09, *P* < 0.001), TC (rho = −0.08, *P* < 0.001), and eGFR (rho = −0.11, *P* < 0.001) and positively correlated with age (rho = 0.07, *P* < 0.001), gender (rho = 0.13, *P *< 0.001), BMI (rho = 0.06, *P* < 0.001), DM (rho = 0.07, *P* < 0.001), hypertension (rho = 0.07, *P* < 0.001), and hyperlipidemia (rho = 0.02, *P* < 0.001) but not significantly correlated with ALT, AST, and asthma (all *P* > 0.05). The results are presented in Supplementary Table S2.

### Logistic regression of SII and SIRI levels and CVD prevalence

Survey-weighted logistic regression was performed to examine the relationship between SII and SIRI levels and the prevalence of CVD among the obese population. However, we found no evidence to suggest that SII was an independent risk factor for the prevalence of CVD (Supplementary Table S3). Additionally, in the crude model, the odds ratios of CVD prevalence increased significantly with the increase in SIRI level, including heart attack, angina, coronary heart disease, congestive heart failure, and stroke in the crude model (*P* for trend <0.001). Moreover, per-SD increase in SIRI levels increased the prevalence of CVD by 35% (1.35 [1.27–1.44]), heart attack by 32% [1.32 (1.24–1.41)], angina by 23% [1.23 (1.14–1.32)], coronary heart disease by 37% [1.37 (1.27–1.47)], congestive heart failure by 41% [1.41 (1.31–1.53)], and stroke by 24% [1.24 (1.16–1.32)], respectively. Besides, this relationship persisted in model 1 after adjusting for demographic variables, including age, gender, ethnicity, education levels, and PIR, except for angina (*P* for trend = 0.067). Furthermore, in models 2 and 3, multivariable regression analysis revealed that SIRI levels were independent risk factors for CVD, including coronary heart disease and congestive heart failure. Moreover, per-SD increase in SIRI was associated with a 13%, 15%, and 28% increase in the risk of CVD (OR = 1.13, 95% CI: 1.04–1.22, *P* = 0.01), coronary heart disease (OR = 1.15, 95% CI: 1.05–1.26, *P* = 0.002), and congestive heart failure (OR = 1.28, 95% CI: 1.16–1.41, *P* < 0.001), respectively, in the corresponding model 3, with maximum adjusted covariates (Table 2).

**Table 2:** Survey-weighted logistic regression examining the association of SIRI with the prevalence of cardiovascular diseases in the obese population.

	*N*	Crude model	Adjusted model 1	Adjusted model 2	Adjusted model 3
		OR (95%CI)	OR (95%CI)	OR (95%CI)	OR (95%CI)
		Cardiovascular diseases			
Q1	376	Reference	Reference	Reference	Reference
Q2	405	1.03 (0.84–1.25)*	1.01 (0.81–1.27)***	0.98 (0.76–1.25)***	0.98 (0.76–1.27)***
Q3	526	1.42 (1.18–1.71)**	1.23 (1.01–1.52)*	1.16 (0.92–1.44)***	1.06 (0.85–1.32)***
Q4	798	2.25 (1.88–2.70)**	1.65 (1.32–2.05)**	1.39 (1.11–1.74)*	1.31 (1.04–1.65)*
*P* for trend		<0.001	<0.001	0.001	0.01
Per SD increase		1.35 (1.27–1.44)**	1.19 (1.11–1.27)**	1.14 (1.06–1.23)**	1.13 (1.04–1.22)*
		Heart attack			
Q1	121	Reference	Reference	Reference	Reference
Q2	161	1.46 (1.05–2.03)*	1.40 (0.97–2.02)***	1.34 (0.91–1.97)***	0.88 (0.64–1.20)***
Q3	201	1.68 (1.22–2.31)*	1.27 (0.89–1.81)***	1.19 (0.82–1.73)***	0.95 (0.66–1.38)***
Q4	345	2.91 (2.20–3.85)**	1.77 (1.28–2.43)**	1.39 (1.00–1.94)***	0.93 (0.67–1.27)***
*P* for trend		<0.001	0.002	0.137	0.395
Per SD increase		1.32 (1.24–1.41)**	1.15 (1.07–1.23)**	1.08 (1.00–1.17)*	1.07 (0.97–1.17)***
		Angina			
Q1	89	Reference	Reference	Reference	Reference
Q2	119	1.80 (1.24–2.62)*	1.66 (1.12–2.46)*	1.59 (1.04–2.42)*	1.59 (1.03–2.45)*
Q3	159	2.16 (1.50–3.11)**	1.69 (1.15–2.49)*	1.50 (1.00–2.26)***	1.32 (0.87–1.99)***
Q4	197	2.75 (1.89–4.0)**	1.69 (1.13–2.51)*	1.38 (0.90–2.10)***	1.23 (0.80–1.91)***
*P* for trend		<0.001	0.067	0.480	0.969
Per SD increase		1.23 (1.14–1.32)**	1.06 (0.96–1.17)***	1.01 (0.90–1.13)***	1.00 (0.88–1.12)***
		Coronary heart disease			
Q1	93	Reference	Reference	Reference	Reference
Q2	128	1.68 (1.18–2.40)*	1.43 (0.97–2.10)***	1.33 (0.88–2.01)***	1.47 (0.94–2.30)***
Q3	195	2.51 (1.83–3.43)**	1.76 (1.26–2.45)*	1.60 (1.12–2.28)*	1.60 (1.11–2.29)*
Q4	304	4.30 (3.17–5.83)**	2.41 (1.71–3.40)**	1.92 (1.35–2.75)**	2.00 (1.35–2.97)**
*P* for trend		<0.001	<0.001	<0.001	0.002
Per SD increase		1.37 (1.27–1.47)**	1.19 (1.10–1.28)**	1.14 (1.05–1.24)*	1.15 (1.05–1.26)*
		Congestive heart failure			
Q1	108	Reference	Reference	Reference	Reference
Q2	107	0.89 (0.62–1.27)***	0.94 (0.64–1.39)***	0.98 (0.63–1.52)***	0.92 (0.57–1.41)***
Q3	176	1.63 (1.13–2.35)*	1.51 (1.01–2.26)*	1.46 (0.97–2.21)***	1.33 (0.86–2.03)***
Q4	306	3.08 (2.23–4.24)**	2.47 (1.70–3.60)**	2.19 (1.49–3.23)**	2.11 (1.39–3.17)**
*P* for trend		<0.001	<0.001	<0.001	<0.001
Per SD increase		1.41 (1.31–1.53)**	1.29 (1.19–1.39)**	1.24 (1.14–1.36)**	1.28 (1.16–1.41)**
		Stroke			
Q1	147	Reference	Reference	Reference	Reference
Q2	135	0.69 (0.50–0.95)***	0.76 (0.54–1.08)***	0.72 (0.49–1.06)***	0.72 (0.48–1.08)***
Q3	173	1.11 (0.82–1.52)***	1.05 (0.74–1.49)***	0.96 (0.67–1.38)***	0.96 (0.64–1.42)***
Q4	281	1.69 (1.28–2.23)**	1.34 (0.95–1.89)***	1.24 (0.86–1.79)***	1.10 (0.75–1.62)***
*P* for trend		<0.001	0.017	0.064	0.248
Per SD increase		1.24 (1.16–1.32)**	1.11 (1.02–1.20)*	1.33 (1.24–1.41)**	1.06 (0.96–1.17)***

SIRI was divided into quartiles, with the lowest group as the reference group.

Model 1 adjust demographic variables including age, gender, Ethnicity, education levels and poverty income ratio.

Model 2 adjust Model 1 plus other parameters and history of diseases including body mass index, estimated glomerular filtration rate, alanine aminotransferase, total cholesterol, and high-density lipoprotein cholesterol, diabetes mellitus and hypertension.

Model 3 adjusted Model 2 plus medication and lifestyle variables including antihypertensives and glucose-lowering drugs, smoking and drinking.

* Indicates *P*-value < 0.05; **indicates *P*-value < 0.001; ***indicates *P*-value ≥ 0.05. SD, standard deviation; OR, odds ratio.

### Subgroup analysis

**Figure 1:**
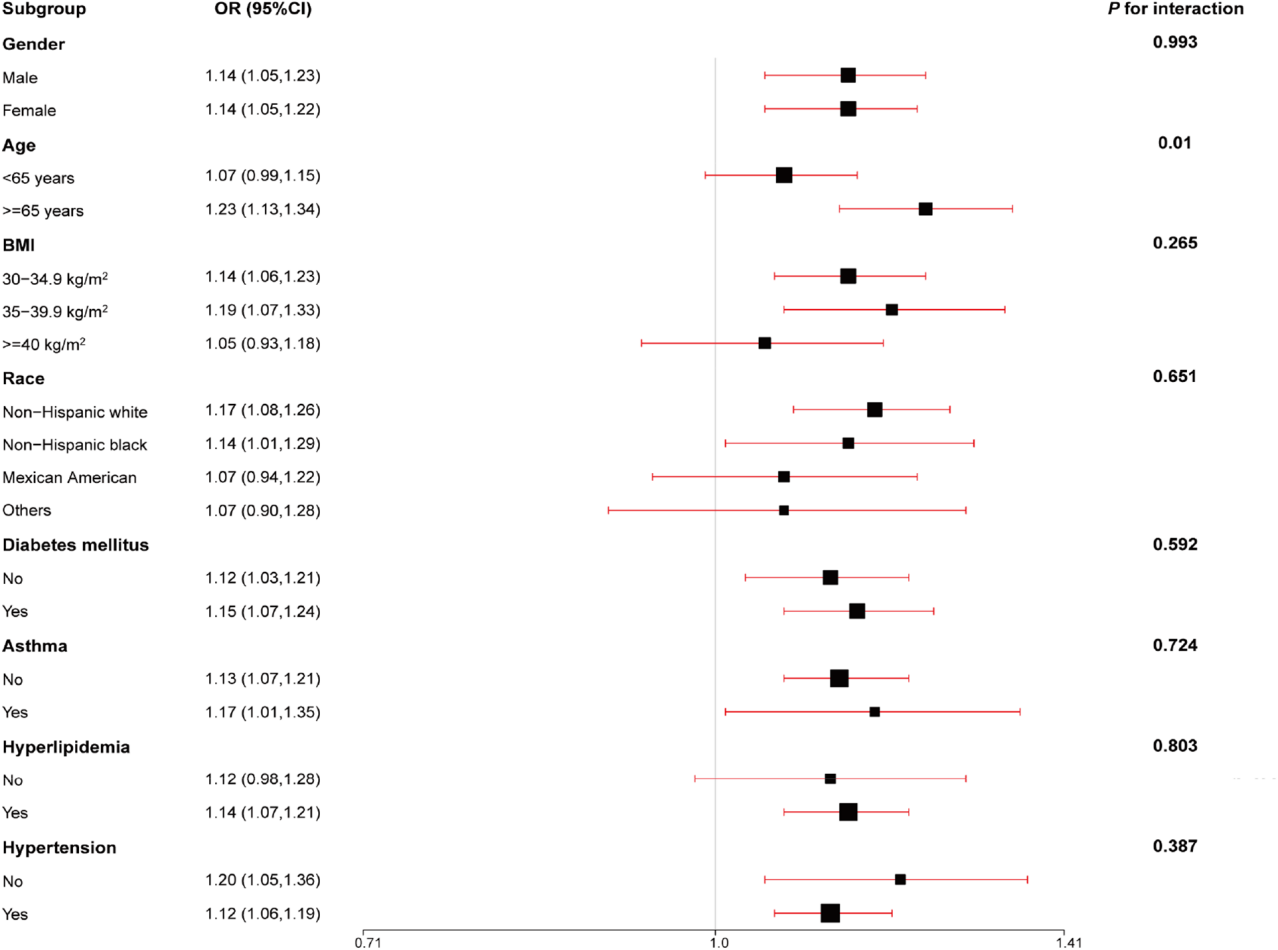
Subgroups were analyzed for the association between an increase in SIRI per one standard deviation and the odds ratios of CVD prevalence, stratified according to different clinical characteristics. The model was adjusted for age, gender, ethnicity, education levels, poverty income ratio (PIR), body mass index (BMI), estimated glomerular filtration rate (eGFR), alanine aminotransferase (ALT), total cholesterol (TC), high-density lipoprotein cholesterol (HDL), diabetes mellitus (DM), hypertension, antihypertensives and glucose-lowering drugs, and smoking and drinking behavior.

Subgroup analyses assessed the performance of SIRI with respect to the prevalence of CVD in some subpopulations. Our findings were robust in most subgroups, including gender, age, BMI, ethnicity, DM, asthma, hyperlipidemia, and hypertension (all *P* for interaction > 0.05). However, the association between SIRI and CVD differed by age in the subgroups (Figure 1). The findings revealed that SIRI was more likely to promote the development of CVD in participants aged ≥65 years (OR = 1.23, 95% CI: 1.13–1.34, *P* = 0.01).

### Predictive value of SIRI

**Figure 2:**
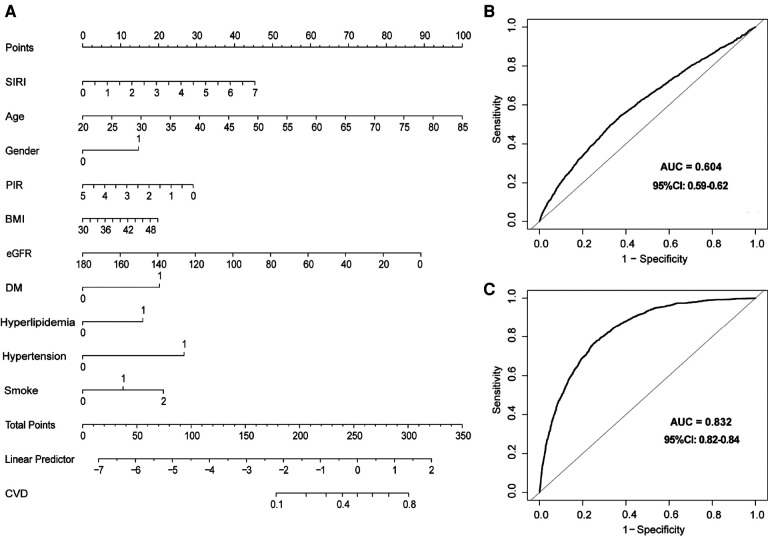
Nomogram and receiver operating characteristics (ROC) curves to evaluate the risk of CVD in participants with obesity. **(A)** Nomogram to predict CVD prevalence. In the DM, hypertension, and hyperlipidemia groups, 0 means “no,” and 1 means “yes”. In the smoking group, 0 means “never”, 1 means “former”, and 2 means “current”. In the gender group, 0 means “female”, and 2 means “male”. **(B)** ROC curve of SIRI for the prevalence of CVD model. **(C)** ROC curves for SIRI combined with clinical variables in the nomogram for the CVD prevalence model. SIRI, systemic inflammation response index; ROC, receiver operating characteristic.

We constructed a nomogram to assess the probability of CVD prevalence using baseline variables that were screened by univariate analysis and multivariate logistic regression models (Supplementary Tables S4 and S5). Finally, ten variables were selected for nomogram construction, including SIRI, age, gender, PIR, BMI, eGFR, DM, hyperlipidemia, hypertension, and smoke. Each variable had a corresponding hazard point, and total points were obtained by summing the points of each variable (Figure 2A).

Moreover, ROC analyses were used to evaluate the predictive value of SIRI in CVD prevalence. SIRI showed a certain accuracy in predicting CVD prevalence (AUC = 0.604, 95% CI: 0.59–0.62) (Figure 2B). In addition, a robust result was revealed when we performed ROC analysis in combination with other variables in the nomogram (AUC = 0.832, 95% CI: 0.82–0.84) (Figure 2C). Finally, calibration curves (Supplementary Figure S1A) and decision curve analysis (DCA) (Supplementary Figure S1B) demonstrated a stable consistency between predicted and actual probabilities.

### Linear association between SIRI and CVD

**Figure 3:**
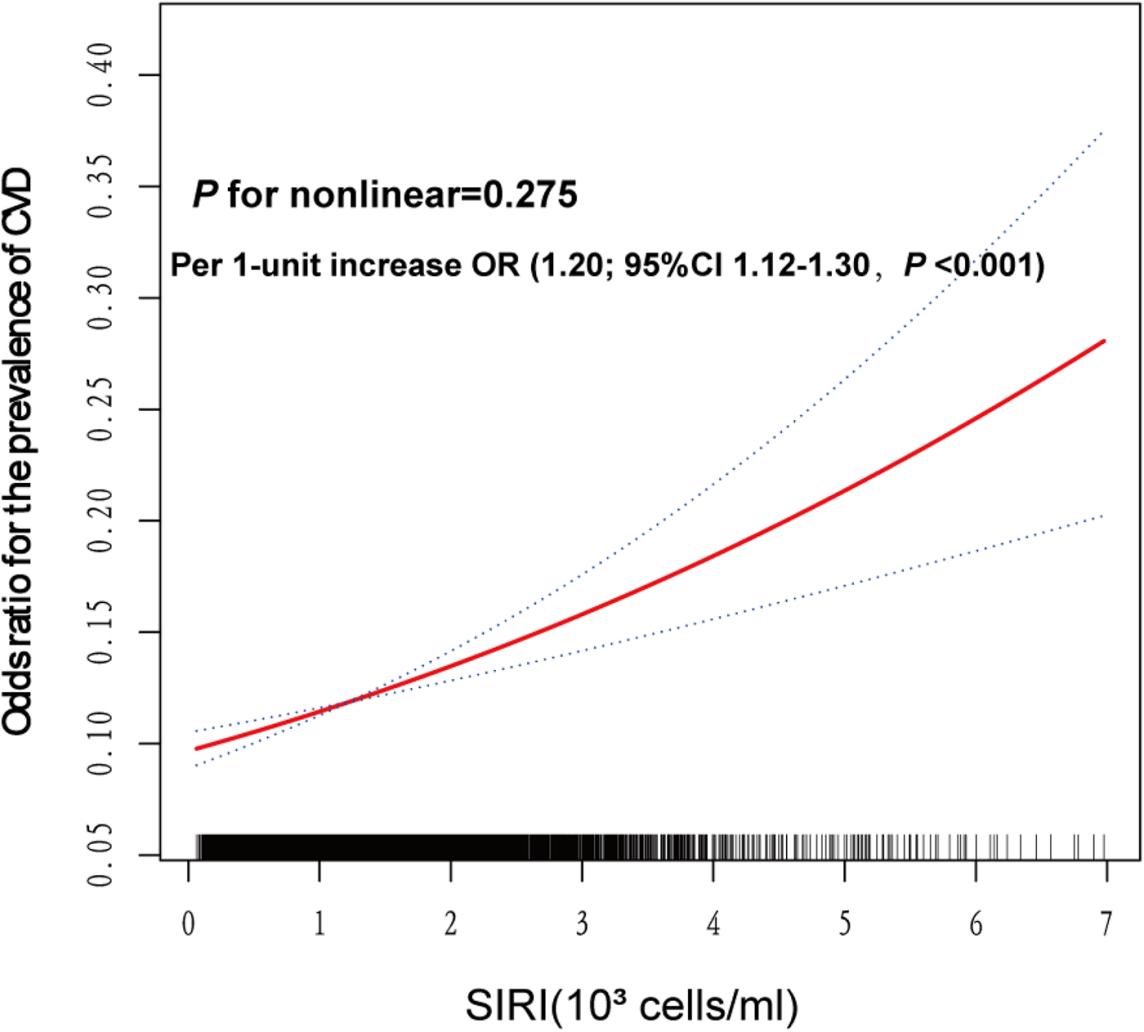
Smooth spline analysis of the relationship between SIRI and the risk of CVD prevalence. Smooth spline analysis was performed through generalized addictive model 3. In the plot, the risk of CVD prevalence increased proportionally with the increment of SIRI, and *P* for nonlinear = 0.275, confirming that the correlation between SIRI and CVD prevalence was linear in the whole range of SIRI.

Smooth curve fitting regression analysis was conducted to estimate the relationship between the odds ratio of SIRI and CVD prevalence based on model 3 (Figure 3). A significant linear association was observed between the odds ratio of SIRI and CVD prevalence (*P* for nonlinear = 0.275). Specifically, for each 1-unit increase in SIRI, the prevalence of CVD increased by 20% (OR = 1.20, 95% CI: 1.12–1.30, *P *< 0.001).

## Discussion

This study is the first cross-sectional study based on participants with obesity to analyze the association between SII, SIRI, and CVD. The study included 17,261 individuals with obesity, and the results can be described as follows, (1) SIRI was an independent risk factor for the prevalence of CVD in participants, whereas SII was not; (2) Higher SIRI values were significantly associated with an increased odds ratio of CVD prevalence; (3) SIRI showed a linear relationship with the risk of CVD prevalence, with a 20% increase in risk per 1-unit increase in SIRI.

Previous studies have explored the association between SII and SIRI and CVD and have investigated the predictive value of these markers using different epidemiological methods. A meta-analysis demonstrated that higher SII was an independent risk factor for the incidence of CVD (HR = 1.39, 95% CI: 1.20–1.61, *P* < 0.001) (26). SII and SIRI were also demonstrated to have a significant value in predicting coronary artery disease and acute coronary syndrome (27). Moreover, a population-based cohort of 85,154 participants in Kailuan exhibited that SII (HR = 1.246, 95% CI: 1.157–1.382) and SIRI (HR = 1.194, 95% CI: 1.087–1.313) were positively associated with an increased risk of stroke (28). However, in our study, we did not observe an association between SII and CVD, including heart attack, angina, coronary heart disease, congestive heart failure, and stroke. The results of univariate cox regression analysis confirmed that SII was not an independent risk factor for the prevalence of CVD. In contrast, SIRI was found to be closely associated with the prevalence of CVD.

Monocytes, neutrophils, and lymphocytes are key components of SIRI (18). Monocytes have a critical role in the inflammatory response and the progression of atherosclerosis (29, 30). Specifically, monocytes migrate to the subendothelium by binding adhesion molecules expressed on the damaged vascular endothelium and gradually mature into macrophages. These cells subsequently bind to class A scavenger receptors (SR-A) and CD-36 to take up oxidized low-density lipoproteins (LDL) and release pro-inflammatory and pro-oxidant cytokines at inflammation sites, causing atherosclerosis (31, 32). Furthermore, the pro-inflammatory and pro-oxidative effects of monocytes are manifested by inhibiting the migration of macrophages and the oxidation of LDL and promoting the accumulation of cholesterol in the vascular wall to lower HDL levels, contributing to the incidence of CVD (33–35).

Neutrophils exhibit a unique defense role in the inflammatory response through degranulation, phagocytosis, production of reactive oxygen species, and construction of neutrophil extethnicityllular traps (NETs) (36). These cytokines activate sterile inflammatory responses in the body, while interactions with vascular endothelial cells and platelets promote immune thrombosis, leading to atherosclerosis and CVD (36, 37). A large cohort study involving 775,231 participants demonstrated that neutrophils counts are closely associated with the incidence of CVD, including heart failure (HR: 2.04, 95% CI: 1.82–2.29), peripheral arterial disease (HR: 1.95, 95% CI: 1.72–2.21), unheralded coronary death (HR: 1.78, 95% CI: 1.51–2.10), abdominal aortic aneurysm (HR: 1.72, 95% CI: 1.34–2.21), and nonfatal myocardial infarction (HR: 1.58, 95% CI: 1.42–1.76) (38).

In contrast, lymphocytes manifest different roles than neutrophils and monocytes in regulating the inflammatory response of the body. *In vivo* experiments also demonstrated that B lymphocyte deficiency promotes the development of atherosclerosis (39). Additionally, clinical studies have shown that low lymphocyte counts are associated with poorer prognosis in patients with CVD, including heart failure, chronic ischemic heart disease, and acute coronary syndrome (40–42). These results suggest the value of SIRI at the cellular level in assessing the inflammatory response of the organism.

A subgroup analysis was performed to estimate if our findings were stable in common subgroups. The survey-weighted logistic regression model was adjusted for all covariates used in model 3. The analysis showed that our findings were robust regarding gender, BMI, ethnicity, DM, asthma, hyperlipidemia, and hypertension. However, a significant difference was found between SIRI and CVD prevalence varied by age. The increase in SIRI may contribute to an increased risk of CVD prevalence in participants aged ≥65 years. Progressive decline in the structure and function of multiple organs are the distinctive features of aging (43). During this complex biological process, vascular remodeling, endothelial dysfunction, and loss of vascular compliance significantly increased the incidence of CVD (44–46). Moreover, aging leads to an increase in fat mass and exhibits chronic low-grade inflammation, as well as is associated with the development of insulin resistance, dyslipidemia, hypertension, and type 2 diabetes mellitus (T2DM) (47, 48). The interaction of these factors increases the risk of CVD prevalence. SIRI is a composite indicator that represents the inflammation in the body. This may explain the exacerbated risk of CVD by SIRI in participants with obesity aged ≥65 years. Similarly, Jun et al. showed that the effect of SIRI on CVD was more likely to be found in participants aged ≥50 years (OR = 1.43, 95% CI: 1.17–1.74) than in those aged <50 years (OR = 1.35, 95% CI: 1.00–1.81) (49). These results indicate that our findings are stable in most populations.

This study was a seminal evaluation of the association of SIRI with CVD prevalence in obese individuals. We revealed a correlation between the dynamic model of SIRI and CVD prevalence, which could be utilized to assess the risk of CVD prevalence in populations with obesity.

However, there are still some limitations to our study. First, because the participants were solely recruited from the United States, our findings can only reflect the situation in this region. Therefore, multicenter investigations are needed in the future. Second, this cross-sectional study lacked follow-up data to demonstrate a causal relationship between SIRI and CVD prevalence or to predict patient outcomes. Third, the U.S. is a diverse country with a population from various ethnic and cultural backgrounds. NHANES assesses the health status and related factors of U.S. permanent residents The NHANES study categorizes race as non-Hispanic black, non-Hispanic white, Mexican American, other Hispanic, and other multiracial. BMI is a global tool used to assess whether an individual is overweight or obese. The cutoff value for obesity may vary due to differences in body composition and health risks among different races. Finally, we did not collect information on participants’ infectious diseases and drugs, which could influence peripheral blood counts and partially affect the results.

## Conclusion

SIRI is independently associated with the prevalence of CVD in individuals with obesity. SIRI demonstrated a better performance in predicting CVD prevalence than SII. Therefore, SIRI could be considered a novel inflammatory indicator to estimate the prevalence of CVD in patients with obesity.

## Data Availability

The original contributions presented in the study are included in the article/Supplementary Materials, further inquiries can be directed to the corresponding author.
