# Supplementary material for: Associations between SII, SIRI, and cardiovascular disease in obese individuals: a nationwide cross-sectional analysis
Source: Front Cardiovasc Med. 2024 Aug 22;11:1361088. doi: 10.3389/fcvm.2024.1361088 (PMC11374596; doi:10.3389/fcvm.2024.1361088)

Supplement

Table S1. survey-weighted baseline characteristics of the obese population in NHANES from 1999 to 2018 according to SII quartiles (N=17,261, representing 68,648,287 individuals with obesity).

Table S2. Correlation between SII, SIRI, and CVD prevalence.

Table S3. Survey-weighted logistic regression examining the association of SII with the prevalence of cardiovascular diseases in the obese population. (SII was divided into quartiles, with the lowest group as the reference group).

Table S4. Differences in baseline information between the CVD and non-CVD groups.

Table S5. Multivariate logistic regression model to assess the relationship between baseline characteristics and CVD prevalence.

Supplemental Figure 1. Calibration and decision curve analysis (DCA) curves of the nomogram model. (A) Calibration curve for the CVD occurrence model. (B) DCA of the CVD occurrence model. CVD, cardiovascular diseases.

**Table S1. survey-weighted baseline characteristics of the obese population in NHANES from 1999 to 2018 according to SII quartiles (N=17,261, representing 68,648,287 individuals with obesity).**					
	Q1 (<352.5)	Q2 (352.5-495.8)	Q3 (495.9-691.4)	Q4 (≥691.5)	*P*-value
Participants number	4,314	4,316	4,315	4,316	
Representing sample size	15,060,168	17,215,814	18,228,638	18,143,667	
**Age (years)**	47.56 (0.36)	46.73 (0.34)	46.17 (0.33)	46.24 (0.34)	0.0064
**PIR**	2.80 (0.04)	2.89 (0.04)	2.93 (0.04)	2.78 (0.04)	0.0010
**BMI(Kg/m^2)**	35.05 (0.10)	35.39 (0.11)	35.93 (0.13)	37.02 (0.14)	<0.0001
**HDL** (mmol/L)	1.23 (0.01)	1.22 (0.01)	1.21 (0.01)	1.24 (0.01)	0.0089
**TC** (mmol/L)	5.08 (0.02	5.10 (0.02	5.10 (0.02	5.07 (0.02)	0.7086
**eGFR**	94.58 (0.46)	95.29 (0.49)	95.37 (0.45)	94.65 (0.54)	0.4021
**ALT (U/L)**	30.33 (0.42)	29.64 (0.37)	28.64 (0.44)	26.69 (0.41)	<0.0001
**AST (U/L)**	27.16 (0.31)	25.73 (0.23)	25.23 (0.30)	24.36 (0.32)	<0.0001
**Gender**					<0.0001
Female	45.57 (43.68 ,47.48)	49.43 (47.38 ,51.48)	52.68 (50.69 ,54.66)	62.24 (60.26 ,64.18)	
Male	54.43 (52.52 ,56.32)	50.57 (48.52 ,52.62)	47.32 (45.34 ,49.31)	37.76 (35.82 ,39.74)	
**Ethnicity**					<0.0001
Non-Hispanic white people	54.75 (51.29 ,58.16)	64.26 (61.31 ,67.11)	67.59 (64.91 ,70.16)	69.71 (66.77 ,72.50)	
Non-Hispanic black people	25.13 (22.39 ,28.08)	14.20 (12.60 ,15.97)	11.02 (9.64 ,12.57)	10.07 (8.78 ,11.52)	
Mexican American	10.18 (8.65 ,11.95)	10.54 (8.98 ,12.34)	10.11 (8.65 ,11.80)	9.78 (8.22 ,11.59)	
Other Ethnicities	9.95 (8.60 ,11.48)	10.99 (9.69 ,12.44)	11.28 (9.89 ,12.84)	10.44 (9.02 ,12.07)	
**Education levels**					0.0009
Less than 9th grade	7.41 (6.57 ,8.36)	6.27 (5.46 ,7.18)	5.75 (5.04 ,6.55)	5.42 (4.70 ,6.24)	
9-11th grade/high school grade or equivalent	38.71 (36.64 ,40.82)	36.56 (34.63 ,38.53)	38.55 (36.49 ,40.65)	40.99 (38.83 ,43.19)	
College graduate or above	53.88 (51.67 ,56.07)	57.17 (55.12 ,59.21)	55.70 (53.42 ,57.97)	53.59 (51.35 ,55.82)	
**Diabetes mellitus**					0.0339
No	80.41 (78.93 ,81.80)	79.20 (77.60 ,80.72)	80.79 (79.21 ,82.28)	77.95 (76.23 ,79.58)	
Yes	19.59 (18.20 ,21.07)	20.80 (19.28 ,22.40)	19.21 (17.72 ,20.79)	22.05 (20.42 ,23.77)	
**Hyperlipidemia**					0.0110
No	22.92 (21.17 ,24.76)	20.15 (18.50 ,21.92)	19.10 (17.60 ,20.70)	19.82 (18.16 ,21.60)	
Yes	77.08 (75.24 ,78.83)	79.85 (78.08 ,81.50)	80.90 (79.30 ,82.40)	80.18 (78.40 ,81.84)	
**Hypertension**					0.2672
No	51.28 (49.16 ,53.39)	52.37 (50.20 ,54.52)	52.20 (50.16 ,54.23)	49.92 (47.97 ,51.86)	
Yes	48.72 (46.61 ,50.84)	47.63 (45.48 ,49.80)	47.80 (45.77 ,49.84)	50.08 (48.14 ,52.03)	
**Asthma**					0.1087
No	87.43 (85.90 ,88.81)	87.19 (85.98 ,88.30)	88.24 (86.98 ,89.39)	86.04 (84.40 ,87.53)	
Yes	12.57 (11.19 ,14.10)	12.81 (11.70 ,14.02)	11.76 (10.61 ,13.02)	13.96 (12.47 ,15.60)	
**Smoking**					0.0089
Never	55.63 (53.54 ,57.69)	56.98 (54.95 ,58.99)	55.92 (53.85 ,57.98)	52.48 (50.21 ,54.74)	

Former	26.00 (24.06 ,28.03)	25.47 (23.87 ,27.15)	24.42 (22.74 ,26.18)	26.37 (24.57 ,28.26)	
Current	18.37 (16.96 ,19.88)	17.55 (16.09 ,19.11)	19.66 (18.07 ,21.35)	21.15 (19.56 ,22.83)	
**Drinking**					0.3449
Never	11.61 (10.38 ,12.96)	13.10 (11.36 ,15.07)	12.36 (10.83 ,14.08)	12.44 (11.07 ,13.94)	
Former	17.19 (15.64 ,18.86)	16.03 (14.45 ,17.75)	16.96 (15.50 ,18.52)	18.37 (16.76 ,20.11)	
Mild/Moderate	33.66 (31.58 ,35.81)	34.22 (31.83 ,36.69)	33.77 (31.45 ,36.17)	31.31 (29.45 ,33.23)	
Heavy	37.54 (35.62 ,39.49)	36.65 (34.67 ,38.67)	36.91 (34.78 ,39.09)	37.88 (35.77 ,40.04)	
**Antihypertensives**					0.0076
No	89.42 (88.11 ,90.61)	89.58 (88.25 ,90.78)	88.25 (86.97 ,89.41)	87.10 (85.79 ,88.31)	
Yes	10.58 (9.39 ,11.89)	10.42 (9.22 ,11.75)	11.75 (10.59 ,13.03)	12.90 (11.69 ,14.21)	
**Glucose-lowering drugs**					0.0651
No	88.16 (86.95 ,89.27)	86.98 (85.66 ,88.20)	87.76 (86.47 ,88.95)	85.99 (84.63 ,87.24)	
Yes	11.84 (10.73 ,13.05)	13.02 (11.80 ,14.34)	12.24 (11.05 ,13.53)	14.01 (12.76 ,15.37)	

Categorical variables were expressed as survey-weighted percentage (95% Confidence interval).

Continuous variables were expressed as survey-weighted mean (Standard Error, SE).

PIR, poverty income ratio, BMI, body mass index. eGFR, estimated glomerular filtration rate. ALT, alanine aminotransferase. AST, aspartate aminotransferase.TC, total cholesterol. HDL, high-density lipoprotein cholesterol. CVD, cardiovascular diseases.

Table S2. Correlation between SII, SIRI, and CVD prevalence

Var1	Var2	Correlation	*P* value	Var1	Var2	Correlation	*P* value	Method
SII	Age	-0.0528	0.0000	SIRI	Age	0.0697	0.0000	Spearman
SII	Gender	-0.1047	0.0000	SIRI	Gender	0.126	0.0000	Spearman
SII	Ethnicity	-0.0646	0.0000	SIRI	Ethnicity	-0.1234	0.0000	Spearman
SII	BMI	0.0975	0.0000	SIRI	BMI	0.0559	0.0000	Spearman
SII	HDL	-0.0078	0.3080	SIRI	HDL	-0.0946	0.0000	Spearman
SII	TC	-0.0097	0.2079	SIRI	TC	-0.0817	0.0000	Spearman
SII	eGFR	0.0129	0.0921	SIRI	eGFR	-0.1117	0.0000	Spearman
SII	ALT	-0.0895	0.0000	SIRI	ALT	0.0124	0.0124	Spearman
SII	AST	-0.128	0.0000	SIRI	AST	-0.0145	-0.0145	Spearman
SII	DM	0.0113	0.1379	SIRI	DM	0.0712	0.0712	Spearman
SII	Hypertension	-0.0046	0.5418	SIRI	Hypertension	0.0699	0.0699	Spearman
SII	Hyperlipidemia	0.0273	0.0003	SIRI	Hyperlipidemia	0.0241	0.0241	Spearman
SII	Asthma	0.003	0.6961	SIRI	Asthma	-0.0037	-0.0037	Spearman

BMI, body mass index. HDL, high-density lipoprotein cholesterol. TC, total cholesterol. eGFR, estimated glomerular filtration rate. ALT, alanine aminotransferase. AST, aspartate aminotransferase. DM, diabetes mellitus. CVD, cardiovascular diseases.

Table S3. Survey-weighted logistic regression examining the association of SII with the prevalence of cardiovascular diseases in the obese population. (SII was divided into quartiles, with the lowest group as the reference group)

	N	Crude Model	Adjusted Model 1	Adjusted Model 2	Adjusted Model 3
		OR (95%CI)	OR (95%CI)	OR (95%CI)	OR (95%CI)
		**CVD**			
Q1	527	Reference			
Q2	469	0.88 (0.72-1.07) [c]	-	-	-
Q3	494	0.88 (0.73-1.06) [c]	-	-	-
Q4	615	1.06 (0.91-1.24) [c]	-	-	-
P for trend		0.38	-	-	-
Per SD increase		1.11 (1.04-1.18) [b]	1.11 (1.03-1.18) [a]	1.08 (0.99-1.17) [c]	-
		**Heart attack**			
Q1	204	Reference	-	-	-
Q2	184	0.96 (0.72-1.27) [c]	-	-	-
Q3	198	0.92 (0.68-1.24) [c]	-	-	-
Q4	242	1.02 (0.79-1.32) [c]	-	-	-
P for trend		0.90	-	-	-
Per SD increase		1.08 (0.99-1.18) [c]	-	-	-
		**Angina**			
Q1	137	Reference	-	-	-
Q2	135	1.08 (0.76-1.53) [c]	-	-	-
Q3	134	0.98 (0.67-1.44) [c]	-	-	-
Q4	158	1.08 (0.82-1.43) [c]	-	-	-
P for trend		0.77	-	-	-
Per SD increase		1.08 (0.98-1.20) [c]	-	-	-
		**Coronary heart disease**			
Q1	182	Reference	-	-	-
Q2	174	0.94 (0.71-1.26) [c]	-	-	-
Q3	170	1.00 (0.74-1.36) [c]	-	-	-
Q4	194	1.05 (0.83-1.33) [c]	-	-	-
P for trend		0.57	-	-	-
Per SD increase		1.10 (1.01-1.20) [a]	1.15 (1.06-1.25) [a]	1.08 (0.96-1.22) [c]	-
		**Congestive heart failure**			
Q1	185	Reference	-	-	-
Q2	143	0.75 (0.55-1.04) [c]	-	-	-
Q3	154	0.80 (0.57-1.14) [c]	-	-	-
Q4	215	1.12 (0.86-1.47) [c]	-	-	-
P for trend		0.28	-	-	-

Per SD increase		1.16 (1.08-1.25) [a]	1.15 (1.06-1.25) [a]	1.15 (1.05-1.27) [a]	1.15 (1.04-1.27) [a]
		**Stroke**			
Q1	171	Reference	-	-	-
Q2	167	1.10 (0.85-1.42) [c]	-	-	-
Q3	166	1.02 (0.76-1.38) [c]	-	-	-
Q4	232	1.28 (0.98-1.67) [c]	-	-	-
P for trend		0.14		-	-
Per SD increase		1.13 (1.05-1.22) [a]	1.10 (1.0-1.19) [a]	1.08 (0.98-1.20) [c]	-

If no significant association was observed, which indicated no independent association of SII with outcome. Subsequent outcomes were then not performed.

[a] **indicates *P*-value<0.05;** [b] **indicates *P*-value<0.001;** [c] **indicates *P*-value≥0.05.** SD, Standard deviation.

Model 1 adjust demographic variables including age, gender, Ethnicity, education levels and poverty income ratio.

Model 2 adjust **Model 1** plus other parameters and history of diseases including body mass index, alanine aminotransferase, alanine aminotransferase, and high-density lipoprotein cholesterol, diabetes mellitus and hyperlipidemia.

Model 3 adjusted **Model 2** plus medication and lifestyle variables including antihypertensives and smoking.

Table S4. Differences in baseline information between the CVD and non-CVD groups.			
	non-CVD	CVD	*P*-value
**Age (years)**	45.6 (45.2 ,45.9)	61.8 (61.1 ,62.5)	<0.0001
**Poverty income ratio**	2.9 (2.9 ,3.0)	2.5 (2.4 ,2.6)	<0.0001
**BMI(Kg/m^2)**	35.8 (35.7 ,36.0)	36.3 (35.9 ,36.7)	0.0134
**HDL** (mmol/L)	1.2 (1.2 ,1.2)	1.2 (1.2 ,1.2)	0.0153
**TC** (mmol/L)	5.1 (5.1 ,5.2)	4.8 (4.7 ,4.9)	<0.0001
**eGFR**	96.4 (95.8 ,97.0)	76.7 (75.5 ,77.9)	<0.0001
**ALT (U/L)**	29.1 (28.6 ,29.5)	26.2 (24.6 ,27.8)	0.0008
**AST (U/L)**	25.6 (25.2 ,25.9)	25.8 (24.6 ,26.9)	0.7320
Gender			0.0070
Female	53.3 (52.2 ,54.3)	48.9 (45.7 ,52.0)	
Male	46.7 (45.7 ,47.8)	51.1 (48.0 ,54.3)	
**Ethnicity**			<0.0001
Non-Hispanic white people	64.0 (61.4 ,66.6)	71.0 (67.9 ,73.8)	
Non-Hispanic black people	14.6 (13.0 ,16.3)	14.8 (12.7 ,17.1)	
Mexican American	10.6 (9.2 ,12.2)	5.1 (4.0 ,6.6)	
Other Ethnicities	10.8 (9.8 ,12.0)	9.1 (7.6 ,11.0)	
**Education levels**			<0.0001
Less than 9th grade	5.7 (5.1 ,6.3)	10.4 (9.1 ,11.9)	
9-11th grade/high school grade or equivalent	37.9 (36.7 ,39.1)	46.0 (42.9 ,49.0)	
College graduate or above	56.4 (55.1 ,57.8)	43.6 (40.6 ,46.8)	
**Diabetes mellitus**			<0.0001
No	82.3 (81.5 ,83.1)	52.2 (49.5 ,54.8)	
Yes	17.7 (16.9 ,18.5)	47.8 (45.2 ,50.5)	
**Hyperlipidemia**			<0.0001
No	21.3 (20.3 ,22.3)	10.2 (8.7 ,12.1)	
Yes	78.7 (77.7 ,79.7)	89.8 (87.9 ,91.3)	
**Hypertension**			<0.0001
No	54.3 (53.1 ,55.5)	19.4 (17.3 ,21.6)	
Yes	45.7 (44.5 ,46.9)	80.6 (78.4 ,82.7)	
**Asthma**			0.7068
No	87.3 (86.5 ,88.1)	86.9 (84.4 ,89.0)	
Yes	12.7 (11.9 ,13.5)	13.1 (11.0 ,15.6)	
**Smoking**			<0.0001
Never	56.8 (55.6 ,58.0)	39.8 (37.0 ,42.6)	
Former	23.9 (22.9 ,25.0)	40.9 (38.0 ,43.9)	
Current	19.3 (18.4 ,20.2)	19.3 (17.2 ,21.5)	
**Drinking**			<0.0001
Never	12.1 (11.0 ,13.3)	12.4 (10.7 ,14.3)	
Former	15.5 (14.6 ,16.5)	32.3 (28.9 ,35.9)	
Mild/Moderate	33.2 (32.0 ,34.5)	34.4 (31.5 ,37.3)	
Heavy	39.1 (38.0 ,40.2)	20.9 (18.4 ,23.7)	
**Antihypertensives**			<0.0001

No	89.5 (88.8 ,90.2)	77.6 (74.9 ,80.1)	
Yes	10.5 (9.8 ,11.2)	22.4 (19.9 ,25.1)	
**Glucose-lowering drugs**			<0.0001
No	89.4 (88.8 ,90.0)	65.7 (63.1 ,68.2)	
Yes	10.6 (10.0 ,11.2)	34.3 (31.8 ,36.9)	

BMI, body mass index. ALT, alanine aminotransferase. AST, aspartate aminotransferase.TC, total cholesterol. HDL, high-density lipoprotein cholesterol. eGFR, estimated glomerular filtration rate. CVD, cardiovascular diseases.

Table S5. Multivariate logistic regression model to assess the relationship between baseline characteristics and CVD prevalence.

	OR	95%CI low	95%CI high	*P*-value
**Age**	1.05491	1.04758	1.06229	<0.000001
**Male**	1.40788	1.17944	1.68056	0.000233
**Ethnicity**				
Non-Hispanic black people	1.16400	0.95210	1.42307	0.140991
Mexican American	0.59735	0.48412	0.73707	0.000004
Other Ethnicities	0.97839	0.74056	1.29260	0.878046
**Educational levels**				
9-11th grade/high school grade or equivalent	1.03264	0.77925	1.36843	0.823422
College graduate or above	0.94215	0.70720	1.25516	0.684561
**PIR**	0.82428	0.78066	0.87034	<0.000001
**BMI**	1.01739	1.00384	1.03112	0.012954
**HDL**	0.83018	0.62614	1.10071	0.198233
**TC**	0.76239	0.69248	1.03936	0.086512
**eGFR**	0.99052	0.98595	0.99511	0.000093
**ALT**	1.00056	0.99797	1.00317	0.671338
**Diabetes mellitus**	1.78622	1.39095	2.29381	0.000012
**Hyperlipidemia**	2.23463	1.73925	2.87110	<0.000001
**Hypertension**	1.84807	1.52936	2.23319	<0.000001
**Smoking**				
Former	1.47286	1.21764	1.78158	0.000111
Current	2.05980	1.65046	2.57067	<0.000001
**Drinking**				
Former	1.47092	1.15468	1.87378	0.002200
Mild/Moderate	1.18057	0.94729	1.47130	0.141880
Heavy	1.07652	0.82421	1.40607	0.589353
**Antihypertensives**	1.05340	0.87224	1.27218	0.589932
**Glucose-lowering drugs**	1.04595	0.80539	1.35837	0.736717
**SIRI**	1.13080	1.02571	1.24664	0.014810

Variables marked in red indicate that they were selected for Nomogram plotting.

BMI, body mass index. ALT, alanine aminotransferase. AST, aspartate aminotransferase.TC, total cholesterol. HDL, high-density lipoprotein cholesterol. eGFR, estimated glomerular filtration rate. CVD, cardiovascular diseases. SIRI, System Inflammation Response Index.

Supplemental Figure 1. Calibration and decision curve analysis (DCA) curves of the nomogram model. (A) Calibration curve for the CVD occurrence model. (B) DCA of the CVD occurrence model. CVD, cardiovascular diseases.

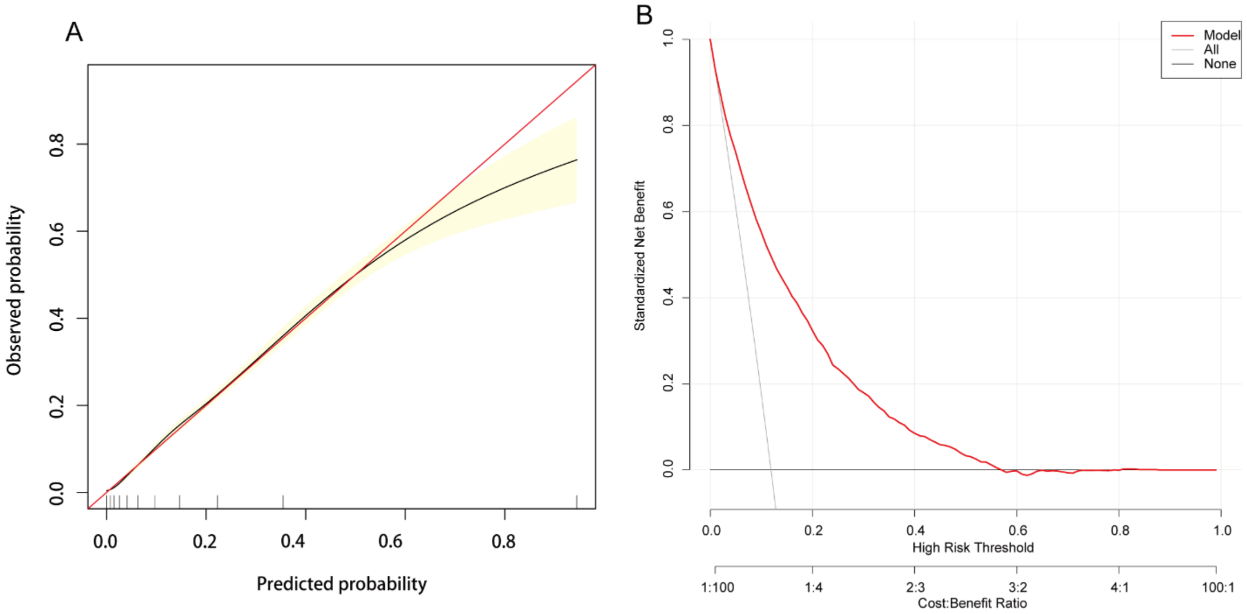

Supplement: Supplementary file 2 [file Datasheet2.pdf]
